# Positive and Negative Relationship between Anxiety and Depression of Patients in Pain: A Bifactor Model Analysis

**DOI:** 10.1371/journal.pone.0047577

**Published:** 2012-10-18

**Authors:** Jingdan Xie, Qian Bi, Wen li, Wen Shang, Ming Yan, Yebing Yang, Danmin Miao, Huiming Zhang

**Affiliations:** 1 Department of Psychology, Fourth Military Medical University, Xi’an, People’s Republic of China; 2 Second Out-Patient Department of the General Logistics Department of PLA, Beijing, People’s Republic of China; 3 State Key Laboratory of Cancer Biology, Xijing Hospital of Digestive Diseases, Fourth Military Medical University, Xi’an, People’s Republic of China; 4 The First Affiliated Hospital of Chinese PLA General Hospital, Beijing, People’s Republic of China; 5 The Health Department of the General Logistics Department of PLA, Beijing, People’s Republic of China; 6 Department of Orthopaedics, Xijing Hospital, Fourth Military Medical University, Xi’an, People’s Republic of China; Federal University of Rio de Janeiro, Brazil

## Abstract

**Background:**

The relationship between anxiety and depression in pain patients has not been clarified comprehensively. Previous research has identified a common factor in anxiety and depression, which may explain why depression and anxiety are strongly correlated. However, the specific clinical features of anxiety and depression seem to pull in opposite directions.

**Objective:**

The purpose of this study is to develop a statistical model of depression and anxiety, based on data from pain patients using Hospital Anxiety and Depression Scale (HADS). This model should account for the positive correlation between depression and anxiety in terms of a general factor and also demonstrate a latent negative correlation between the specific factors underlying depression and anxiety.

**Methods:**

The anxiety and depression symptoms of pain patients were evaluated using the HADS and the severity of their pain was assessed with the visual analogue scale (VAS). We developed a hierarchical model of the data using an IRT method called bifactor analysis. In addition, we tested this hierarchical model with model fit comparisons with unidimensional, bidimensional, and tridimensional models. The correlations among anxiety, depression, and pain severity were compared, based on both the bidimensional model and our hierarchical model.

**Results:**

The bidimensional model analysis found that there was a large positive correlation between anxiety and depression (*r* = 0.638), and both scores were significantly positively correlated with pain severity. After extracting general factor of distress using bifactor analysis, the specific factors underlying anxiety and depression were weakly but significantly negatively correlated (*r* = −0.245) and only the general factor was significantly correlated with pain severity. Compared with the three first-order models, the bifactor hierarchical model had the best model fit.

**Conclusion:**

Our results support the hypothesis that apart from distress, anxiety and depression are inversely correlated. This finding has not been convincingly demonstrated in previous research.

## Introduction

Pain often co-occurs with negative psychological moods, especially depression and anxiety. Arnold et al. [1] found that depression and anxiety were alleviated to a certain extent while treating fibromyalgia with pregabalin, and the change in pain showed a low to moderate positive correlation with the change in depression and anxiety. Kuijpers et al. [2] applied the self-report Hospital Anxiety and Depression Scale (HADS) to 344 patients with noncardiac chest pain and found that 266 patients’ depression and anxiety scores were equal to or higher than 8, the cut-off score used to screen for depressive and anxiety disorders. An additional diagnosis with the Mini International Neuropsychiatric Interview revealed that 198 patients met the diagnostic criteria for panic disorder or depression. In addition, numerous studies have shown a relatively high correlation between these psychological symptoms. Bossala et al. [3] found that the correlation of anxiety and depression reached 0.77 among patients with chronic hemodialysis.

The HADS is a common tool used to screen for anxiety and depression in patients with bodily diseases [4]. The research of Kuijpers et al. [2] indicates that this scale is an adequate screening tool for certain patient populations. Therefore, this simple scale has been widely applied to the psychological assessment of many non-psychiatric patients.

The original construct of the scale put forward by Zigmond and Snaith [4] was a two-factor (bidimensional) model in which the items in the depression and anxiety sub-scales were thought to measure the factors of depression and anxiety respectively. However, many researchers have questioned the constructs of this scale [5] using unidimensional [6], bidimensional [7], and tridimensional models [8] based on classical testing theory (CTT) as well as latent construct analysis based on non-parametric Item Response Theory (IRT) [9] and unidimensional IRT [10]–[13]. These results indicated that additional analyses on the constructs of this scale are necessary to ensure its reliability and validity in clinical application. Further, while determining the applicability of the HADS to a Spanish population, Herrero et al. [14] found that there were significant correlations among all 14 items, the two subscale scores, and the total score. This finding indicates that all these items can reflect characteristics of both anxiety and depression to some extent. Hence each item has multidimensional characteristics and cannot be assigned to a unidimensional scale corresponding to either of the sub-scale scores or the total score. Thus, a multidimensional model should be adopted to obtain more accurate results using the HADS.

With the development of psychometrics and, in particular, the application of multidimensional IRT models (e.g., bifactor models [15]) to clinical psychological assessments, researchers have obtained a new understanding of the latent constructs underlying clinical psychological symptoms [16]. Further, there have been new discoveries regarding these latent constructs and the relationship between anxiety and depression. Simms et al. [17] analyzed the Inventory of Depression and Anxiety Symptom (IDAS) using bifactor analysis and found that many manifestations of depression and anxiety primarily reflect the influence of a single general factor of psychological distress, whereas other manifestations reflect the effects of both general and specific factors. Simms et al. identified one general factor (distress) and 13 specific factors responsible for different clinical manifestations of distress as depressive or anxiety symptoms. This model is hierarchical in that the general factor is placed at a higher-order of abstraction, while all the specific factors reside at a lower level governed by the general factor. Given this research, we must consider whether there are similar latent constructs with regard to anxiety and depression for patients in pain. Only with a precise understanding of this issue can we achieve accurate measurement and effective intervention.

Since the HADS is not as fine grained as the IDAS used in Simms et al. [17], our hierarchical model has only two specific factors, one for depression and one for anxiety. In this respect, our model is structurally similar to the tripartite hierarchical model suggested by Dunbar et al. [8], although they used a different statistical methodology to construct their model (confirmatory factor analysis). Therefore, we call our bifactor model, the tripartite hierarchical model, or hierarchical model for short.

As the main psychological disorders in pain patients, both anxiety and depression have a distress component, which may reflect the positive correlation between them. On the other hand, analyzed from the perspective of psychomotor theory, anxiety motivates action, and can prompt people to deal with problems. In contrast, depression inhibits action and can lead to avolition. These considerations led us to hypothesize that beneath the strong positive relationship mediated by general distress, anxiety and depression might actually be negatively related.

In order to tease apart the positive and negative relationships between depression and anxiety we developed a hierarchical model that separated the general distress component of both types of symptoms from their specific characteristics. Moreover, this research compared the statistical relationships between anxiety, depression and patient pain severity as analyzed based on the original two-factor HADS construct against those revealed using the hierarchical model. We also tested our model’s fit to the data in comparison with unidimensional, bidimensional, and tridimensional models.

## Methods

### Ethics Statement

This study was approved by the Ethics Committee of the Fourth Military Medical University and the Second Out-Patient Department of the General Logistics Department of the People’s Libration Army (PLA) in China. All the adult patients gave their written informed consent to agree to participate in the investigation. As for child patients, defined as those younger than 16, written informed consent was obtained from the patients themselves as well as their adult guardians (signing on the same consent paper).

### Participants

In cooperation with many medical centers, we surveyed patients in pain from 10 medical institutions across four cities in China: Beijing, Xi’an, Chongqing, and Lanzhou. These patients were treated in seven different departments, including the pain clinic and the departments of orthopedics, cardiology, gastroenterology, neurology, stomatology, and oncology. Doctors and nurses assessed participant pain symptoms while outpatients were waiting for treatment. All these patients were then randomly selected to be surveyed. All investigators had been trained in psychological assessment. The investigators explained the purpose of the survey to the patients and responded to questions and concerns in order to foster trust and cooperation and to obtain written informed consent.

Investigators screened patients informally to determine if they had adequate cognitive ability to understand the meaning of the survey. We did not have enough time to conduct tests to formally measure the cognitive abilities of patients. Investigators only included a patient in the study if the patient could readily understand the meaning of the survey and there was little difficulty communicating with them. Those that were evaluated as having adequate cognitive abilities to participate in the study were designated as having “normal intelligence.” Otherwise, they were designated as “Does not have appropriate intelligence to complete the survey.”

A specially assigned person distributed the survey questionnaires in order to ensure confidentiality and voluntary participation. If patients had questions when completing the survey, they consulted the investigators. The investigators thanked participants when they finished the questionnaire.

#### Participant inclusion criteria

Patients designated as having “normal intelligence” who were able to understand the content of the questionnaire and were capable of completing the tests by themselves or with the assistance of investigators were included in this study. Participants also had to be free from organic brain damage and infection and could not have previously accepted treatment for their psychological symptoms.

#### Participant exclusion criteria

Patients designated as “Does not have appropriate intelligence to complete the survey” or were otherwise unable to complete the questionnaire effectively due to physical disease, organic brain damage or infection, or other reasons were excluded from the study.

Ultimately, 535 patients with symptoms of pain were selected for this research. These participants were from 11 provinces, cities, and autonomous regions in China, including Shaanxi, Gansu, Shanxi, Beijing, Chongqing, and so on. We excluded data from respondents with many missing values; thus, 503 valid surveys were collected (52.7% male and 47.3% female). Their ages ranged from 16 to 91 years old (mean = 47.21 years, standard deviation = 16.76 years). At first, we had participants who were under 16, but all those surveys were excluded because of too many missing values. Among the valid surveys, 42 patients had cancer, 252 patients had chronic spinal pain, 132 patients had an acute exacerbation of chronic pain (e.g., coronary atherosclerotic heart disease, trigeminal neuralgia and so on), and 77 patients had acute pain (e.g., fractures, sprains, and so forth). In this study, chronic pain refers to symptoms that are related to their diseases that have been present for more than 6 months.

### Materials

A demographic questionnaire collected data regarding patient age, sex, and medical status. The Chinese version of the HADS investigated the anxiety and depression of patients in pain. This scale contains 14 items, of 7 form an anxiety subscale and 7 form a depression subscale. Each item consists of 4 severity levels (0, 1, 2, 3). Previous research using the Chinese version of this scale with cardiology, endocrinology, and end-stage renal failure patients revealed that a cut-off value of 9 points for either the anxiety or depression subscales could screen anxiety and depression well [18].

To measure pain severity for the purpose of assessing the correlation between pain and depression/anxiety symptoms, this study adopted the visual analogue scale (VAS). The VAS is a simple and effective pain assessment with high sensitivity that measures pain severity linearly. The “0” at the left end represents no pain, and the “10” at the right end represents severe and unbearable pain. In a clinical assessment, a score less than 2 points is “excellent,” 3–5 points is “good,” 6–8 points is “average,” and over 8 points is “poor.” Other factors seldom influence this assessment of pain. Thus, it is widely used in initial clinical assessment and has been validated in Chinese clinical populations [19].

### Analyses

CTT and IRT bifactor analyses were conducted on the HADS results, and the bifactor analysis was tested in a model fit comparison. For the CTT analysis we calculated two subscale scores according to the original two-factor construct of HADS, and conducted descriptive analyses of the two subscale scores. We then compared distributions of anxiety and depression disorders in different diseases using chi-square tests. And the correlations between items and the subscale scores, as well as overall score, and the internal consistency coefficients of the two subscales (Cronbach’s alpha) were analyzed.

**Figure 1:**
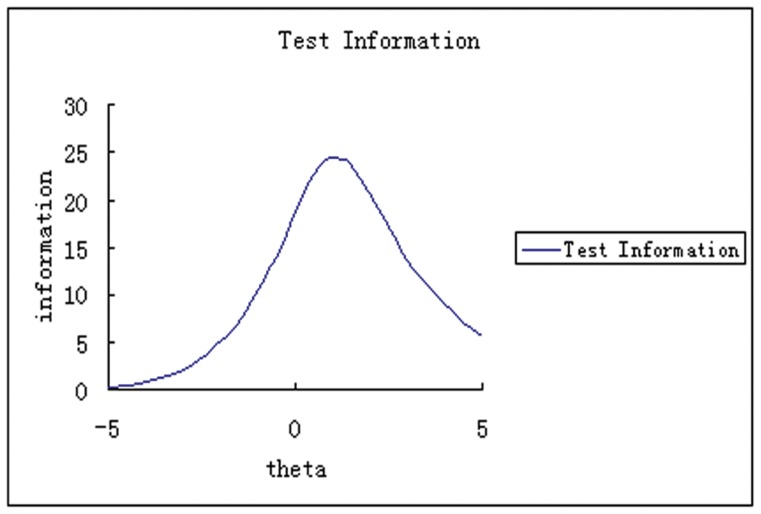
The test information curve of the HADS based on the bifactor analysis for the general distress factor. X-axis represents severity of the general factor (theta), which had been standardized (0 being average, 1 being a standard deviation). The Y-axis represents the test information value. Test information is a kind of reliability criterion in IRT models, the bigger the test information value, the less measurement error, and better reliability. In contrast to models built using CTT, in IRT models, there is a test information value corresponding to every severity point, representing the reliability at that level of severity. We get the test information curve by connecting all these values.

Bifactor analysis is a kind of IRT modeling that allows the assessment of direct fit for hierarchical models in which a general factor is separated from several specific factors [17]. Bifactor analysis can also provide us with standardized subject scores (0 being average and 1 being standard deviation, Figure 1) and main parameters for each item as well as the whole scale (Table 1). Factor loading has the same meaning as loadings in other kinds of factor analyses. Slope is a kind of discrimination parameter. Items with higher slopes are better at discriminating between patients with symptoms of different severity. The severity parameter is a kind of the location parameter. A larger severity parameter represents more severe symptoms. Test information is a kind of reliability criterion. Larger test information represents more accurate results. Unlike in classical testing theory (CTT), in which reliability for a scale is just one value, test information is a kind of function, with severity being X-axis, and information value being Y-axis. So we could know on what severity, the scale can get most accurate results.

**Table 1:** Main parameters of bifactor analysis.

ITEMS	loading 1^a^	loading 2^b^	loading 3^c^	slope 1^d^	slope 2^e^	slope 3^f^	severity 1^g^	severity 2^h^	severity 3^i^
ITEM1	0.482	0.43	–	0.632	0.564	–	−1.253	0.119	0.993
ITEM2	0.345	–	0.252	0.381	–	0.279	−0.437	0.771	1.264
ITEM3	0.611	0.427	–	0.915	0.640	–	−0.913	−0.196	0.960
ITEM4	0.417	–	0.564	0.585	–	0.791	−0.254	0.576	1.338
ITEM5	0.771	0.204	–	1.280	0.339	–	−0.517	0.374	1.162
ITEM6	0.793	–	0.092	1.316	–	0.153	−0.563	0.348	1.358
ITEM7	0.347	−0.001	–	0.370	−0.001	–	−1.058	0.129	1.517
ITEM8	0.552	–	0.005	0.661	–	0.006	−0.497	0.081	1.139
ITEM9	0.385	0.144	–	0.422	0.158	–	−1.077	0.142	1.044
ITEM10	0.567	–	0.445	0.819	–	0.642	−0.083	0.916	1.528
ITEM11	0.710	0.214	–	1.058	0.319	–	−0.793	0.332	1.350
ITEM12	0.803	–	−0.045	1.351	–	−0.075	−1.090	0.411	1.529
ITEM13	0.462	0.038	–	0.522	0.043	–	−0.635	0.741	1.730
ITEM14	0.454	–	0.394	0.568	–	0.493	−0.442	0.508	1.112

Loading 1^a^: factor loading on general factor.

Loading 2^b^: factor loading on anxiety specific factor.

Loading 3^c^: factor loading on depression specific factor.

Slope 1^d^: item slop of general factor, a kind of discrimination parameter for general factor.

Slope 2^e^: slopes of anxiety specific factor, a kind of discrimination parameter for anxiety specific factor.

Slope 3^f^: slopes of depression specific factor, a kind of discrimination parameter for depression specific factor.

Severity 1^g^: boundary severity of general factor from score 0 to 1.

Severity 2^h^: boundary severity of general factor from score 1 to 2.

Severity 3^i^: boundary severity of general factor from score 2 to 3.

**Figure 2:**
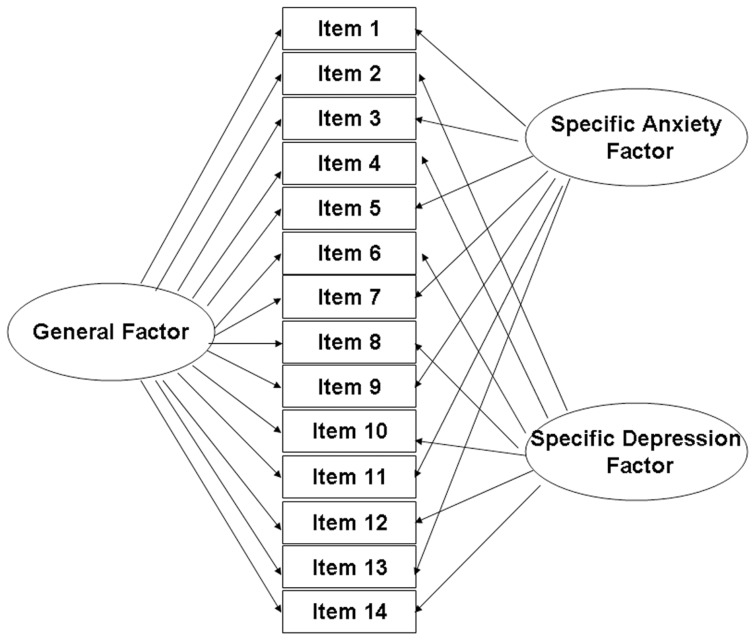
Structure of the hierarchical model of the HADS built using bifactor analysis. In the original scale, 14 items load on 2 subscales (anxiety and depression) respectively. In which, item 1, 3, 5, 7, 9, 11, and 13 belong to anxiety subscale. And item 2, 4, 6, 8, 10, 12, and 14 belong to depression subscale. In the bifactor analysis, all the items have loadings on both the general distress factor and one of the subscale specific factors.

In addition to performing CTT bidimensional and IRT bifactor analyses of the HADS data, we wanted to prove the superiority of the hierarchical model created through the IRT bifactor analysis. To this end, we conducted several confirmatory factor analyses to compare the model fit of different models. Guided by the systematic review of the latent structure of HADS [5], we chose three representative competing models: unidimensional [6], bidimensional, and tridimensional [8]. The bidimensional model was in accordance with the original construct of the scale, while the tridimensional model followed Clark and Watson’s [20] suggestion for a non-hierarchical three-factor model. Our own tripartite hierarchical model, constructed using bifactor analysis, can be seen as a combination of the unidimensional and bidimensional models (see Figure 2).

Lastly, correlations between pain severity and psychological factors were calculated. In the bidimensional (original construct) analysis, we correlated pain severity with depression and anxiety scores, while in the bifactor analysis we correlated pain severity with general distress and the two specific factors underlying depression and anxiety. All the CTT analyses, with the exception of the confirmatory factor analyses, were conducted using SPSS for Windows 13.0. The model fit comparison was conducted based on confirmatory factor analyses completed using Lisrel 8.50. Finally, Polybif software [21] was employed to calculate the standardized scores of the general factor and the two specific factors, as well as parameters for each item.

## Results

### CTT Analyses of the HADS

In the original construct of HADS, there are two subscales corresponding to two factors: anxiety and depression. First, we calculated the internal consistency (Cronbach’s alpha) of each subscale. The results showed that for anxiety α = 0.734 and depression α = 0.719, which were both acceptable. Descriptive analyses of the two subscale scores revealed that the average anxiety score was 9.3, and the standard deviation was 3.9; the average depression score was 7.6, and the standard deviation was 4.0. Table 2 shows the proportions of different diseases for which anxiety or depression scores were greater than 9 points, which is the screening cut-off score for anxiety and depression subscales in the Chinese edition of the HADS [18]. Chi-square tests showed no significant difference in the rates of depression or anxiety between different medical conditions.

**Table 2:** Distributions of anxiety and depression disorders in different diseases.

	Cancer (n = 42)	Spinal disease chronic pain(n = 252)	Acute exacerbation of chronic pain (n = 132)	Acute pain (n = 77)	*χ^2^*
Anxiety≥9	28(67%)	153(61%)	79(60%)	46(60%)	0.69
Depression≥9	19(45%)	106(42%)	60(45%)	38(49%)	1.39

* *p*<0.05.

To further analyze patient response features, we calculated the correlations of the item, subscale and total scores. The results were in accordance with Herrero et al. [14]. Table 3 shows that significant correlations exist between each survey item and the HADS anxiety and depression subscales. This finding means that these items have multidimensional features; thus, a multidimensional model should be adopted in this analysis. There was also a relatively high correlation between anxiety and depression subscale scores (*r* = 0.638, *p*<0.001). In addition, descriptive analyses of the VAS pain severity assessment revealed that the average pain severity value was 4.9 out of 10, and the standard deviation was 2.4.

**Table 3:** Correlations between items and HADS scales.

Item	Anxiety subscale	Depression subscale	Full scale
A1 (Item1: Tense)	**0.663** **	0.378**	0.576**
A2 (Item3: Frightened)	**0.712** **	0.403**	0.611**
A3 (Item5: Worrying thought)	**0.767** **	0.549**	0.734**
A4 (Item7: Feel relaxed)	**0.404** **	0.322**	0.383**
A5 (Item9: “Butterflies” in the stomach)	**0.503** **	0.234**	0.409**
A6 (Item11: Restless)	**0.707** **	0.488**	0.661**
A7 (Item13: Panic attack)	**0.512** **	0.375**	0.489**
D1 (Item2: Enjoy the things I used to)	0.247**	**0.479** **	0.393**
D2 (Item4: Laugh and see the funny side)	0.302**	**0.611** **	0.489**
D3 (Item6: Cheerful)	0.588**	**0.711** **	0.713**
D4 (Item8: Slowed down)	0.405**	**0.607** **	0.564**
D5 (Item10: Lost interest in appearance)	0.396**	**0.670** **	0.582**
D6 (Item12: Look forward with enjoyment)	0.604**	**0.641** **	0.696**
D7 (Item14: Enjoy a good book, radio or TV)	0.362**	**0.611** **	0.533**

In anxiety and depression subscales, the highest value for each item is shown in bold type.

Definition of each item is the same with Herrero [14].

** All correlations are significant at the 0.01 level or above.

### IRT Bifactor Analyses of the HADS

In this study we found that, except for the fourth item, “Laugh and see the funny side”, the general distress factor loadings were larger than the specific (anxiety/depression) factor loadings. This finding suggests that these 13 items measure the severity of the general factor (i.e., psychological distress) more than those of the specific factors. Test information values indicate that the HADS accurately measured the general factor for a large range of severity of the general factor, but less accurately for patients with very low or very high severity (Figure 1). Interestingly, we found that the specific factors of anxiety and depression are significantly negatively correlated (*r* = −0.245, *p*<0.001). Although the correlation coefficient is not so great, it is very valuable when we notice that when combined with general distress factor, the correlation was 0.638 for anxiety and depression. This study for the first time shows the negative correlation between specific factors of anxiety and depression.

To compare the influence of the different analyses to the assessment results and prove the validity of the bifactor analysis with regard to the clinical evidence, we calculated the correlations between the psychological symptoms and pain severity using a bidimensional model and our bifactor analyses. The results showed that low but significant correlations exist between pain severity and anxiety as well as depression scores under the bidimensional model (*r*_anxiety_ = 0.205, *p*_anxiety_<0.001; *r*_depression_ = 0.163, *p*_anxiety_<0.001). Using our hierarchical model, distress scores and pain severity were weakly but significantly correlated (*r*_distress_ = 0.195, *p*_distress_<0.001), whereas specific factors of anxiety and depression were not significantly correlated with pain severity.

As for the low strength of the correlations, there are several possible reasons. First, anxiety and depression symptoms of patients could be influenced by many variables other than pain severity, such as their social support, coping styles, interpersonal relationship, etc. Second, although the sample size was relatively large, there were some patients whose pain severities were extremely low or high and from the results above, we know that HADS scores are less accurate for extreme subjects. Third, by comparing our results with previous research [1], we found reason to believe that the findings of low but significant correlations between depression/anxiety and pain severity were relatively reliable.

### Model Comparison

In order to provide evidence for the superiority of our hierarchical model, we conducted a model fit comparison between unidimensional, bidimensional, tridimensional, and hierarchical models. The model constructs have been explained in the Analyses section above. As shown in Table 4, the hierarchical model had the best model fit indices, including CFI, GFI and RMSEA. Also, we can see that changes of chi-squares are all significant when we compare other three models with hierarchical model.

**Table 4:** Model fit comparison between unidimensional, bidimensional, tridimensional, and hierarchical models.

	CFI	GFI	RMSEA	*χ^2^*	*df*	*△χ^2^*	*△df*
Unidimensional	0.83	0.86	0.11	595.39	77	385.24*	17
Bidimensional	0.84	0.87	0.11	555.94	76	345.79*	16
Tridimensional	0.84	0.87	0.11	458.37	74	248.22*	14
Hierarchical	0.94	0.94	0.07	210.15	60	–	–

* *p*<0.05.

*△χ^2^* and *△df* represent model fit comparison between unidimensional, bidimensional, tridimensional models and hierarchical model.

## Discussion

Anxiety and depression often co-occur with pain [22]. However, according to psychodynamics and clinical observation of patient symptoms, anxiety motivates action, and may sometimes encourage people to work toward solving their problems. Depression, on the other hand, features behavioral inhibition, and tends to undermine action. Traditional (first-order) statistical models cannot reflect these features since they show anxiety and depression to be positively correlated. The current research used bifactor analysis to build a hierarchical model of depression/anxiety to show, for the first time, that anxiety and depression may correlate positively because of the existence of a general factor of psychological distress, while at the same time also displaying a negative correlation between their specific features.

Likewise for understanding the relationship between pain and depression/anxiety, we found that using the original two-factor construct, both anxiety and depression were significantly correlated with pain severity. However, using our hierarchical model, we showed that the general factor of distress accounts completely for the link between pain and depression/anxiety.

Simms et al. [17] and other researchers have concluded that the common factor underlying diverse anxiety and depression symptoms is psychological distress. The present study has furthered our understanding of this general distress factor by showing that it mediates the relationship between depression/anxiety and pain. That is to say, the aspect of anxiety and depression that interacts with a patients’ degree of physical pain is distress. This finding could help us to better conceptualize the relationship between physical pain and psychological symptoms, and ultimately, might be useful to help health professionals alleviate both more effectively. It is possible that the positive correlations between pain alleviation and depression/anxiety reduction found in Arnold et al. [1] could be explained by the fact that pain treatment led to a reduction in psychological distress. This hypothesis could be tested explicitly in future research.

As discussed in Herrero et al. [14], the structure of the HADS has been a popular topic. Although many items load strongly on the general factor, some items (e.g., tense, frightened, and so on) also have good loadings on specific factors. Therefore, a bifactor model might describe the construct of the HADS more precisely than other models. This precision might demonstrate global distress as well as reflect specific factors of anxiety and depression. This construct feature might explain the production of poor ROC curves when the total score is applied to demonstrate psychological symptoms [23].

This research has a number of obvious shortcomings. First, diagnostic interviews were not applied to determine whether patients had mental disorders, and screening cut-off values for the general factor and the specific factors were not defined with an ROC curve. Second, the sample size was not large. Although there were various disease types included in this research, the number of patients with each kind of disease was small, and the characteristics of distress for each kind of disease was not explored in depth. As a result, although we found interesting correlations between depression/anxiety and pain severity, the correlations were a bit low.

Analyzing clinical assessments with complex mathematical models might reveal their strengths and weaknesses more clearly, increasing the accuracy of theoretical and practical measurement and advancing psychological theories. This research provides an example of how the powerful techniques of IRT can be applied to a well-known clinical measurement tool. Our results show that a hierarchical model built using bifactor analysis can describe the relationship between anxiety and depression in pain patients more precisely and accurately than first-order models built using CTT. In addition, bifactor analysis allows us to determine that the distribution of severity parameters for all the items was even and relatively limited in range. However, our results showed that the capacity of the HADS to measure low and high extremes of distress was limited. To promote the clinical efficacy of the HADS in screening non-psychiatric patients’ for psychological symptoms, additional items to measure low and high extremes of distress should be added. Moreover, in situations where computerized adaptive testing technique can be applied, then the accuracy and convenience of the HADS in clinical application will be improved.
